# Glutamate Chemical Exchange Saturation Transfer (GluCEST) Magnetic Resonance Imaging in Pre-clinical and Clinical Applications for Encephalitis

**DOI:** 10.3389/fnins.2020.00750

**Published:** 2020-07-28

**Authors:** Yanlong Jia, Yanzi Chen, Kuan Geng, Yan Cheng, Yan Li, Jinming Qiu, Huaidong Huang, Runrun Wang, Yunping Zhang, Renhua Wu

**Affiliations:** ^1^Department of Radiology, The Second Affiliated Hospital of Shantou University Medical College, Shantou, China; ^2^Department of Radiology, Affiliated Longhua People’s Hospital, Southern Medical University, Shenzhen, China; ^3^Department of Radiology, The First People’s Hospital of Honghe Prefecture, Mengzi, China; ^4^Department of Nuclear Medicine, Shenzhen Luohu District People’s Hospital, Shenzhen, China

**Keywords:** chemical exchange saturation transfer, encephalitis, *Staphylococcus aureus*, glutamate, magnetic resonance imaging

## Abstract

**Background:**

Encephalitis is a common central nervous system inflammatory disease that seriously endangers human health owing to the lack of effective diagnostic methods, which leads to a high rate of misdiagnosis and mortality. Glutamate is implicated closely in microglial activation, and activated microglia are key players in encephalitis. Hence, using glutamate chemical exchange saturation transfer (GluCEST) imaging for the early diagnosis of encephalitis holds promise.

**Methods:**

The sensitivity of GluCEST imaging with different concentrations of glutamate and other major metabolites in the brain was validated in phantoms. Twenty-seven Sprague–Dawley (SD) rats with encephalitis induced by *Staphylococcus aureus* infection were used for preclinical research of GluCEST imaging in a 7.0-Tesla scanner. For the clinical study, six patients with encephalitis, six patients with lacunar infarction, and six healthy volunteers underwent GluCEST imaging in a 3.0-Tesla scanner.

**Results:**

The number of amine protons on glutamate that had a chemical shift of 3.0 ppm away from bulk water and the signal intensity of GluCEST were concentration-dependent. Under physiological conditions, glutamate is the main contributor to the GluCEST signal. Compared with normal tissue, in both rats and patients with encephalitis, the encephalitis areas demonstrated a hyper-intense GluCEST signal, while the lacunar infarction had a decreased GluCEST signal intensity. After intravenous immunoglobulin therapy, patients with encephalitis lesions showed a decrease in GluCEST signal, and the results were significantly different from the pre-treatment signal (1.34 ± 0.31 vs 5.0 ± 0.27%, respectively; *p* = 0.000).

**Conclusion:**

Glutamate plays a role in encephalitis, and the GluCEST imaging signal has potential as an *in vivo* imaging biomarker for the early diagnosis of encephalitis. GluCEST will provide new insight into encephalitis and help improve the differential diagnosis of brain disorders.

## Introduction

Encephalitis is serious inflammatory disease of the central nervous system (CNS) that is caused by many physiological or pathological factors (Kumar, 2020). According to its etiology, encephalitis can be divided into three types, namely, infectious encephalitis (e.g., bacterial, viral, fungal, and tuberculosis), autoimmune encephalitis, and unexplained encephalitis. Encephalitis is mainly caused by the degeneration or necrosis of neurons by the invading brain parenchyma. Owing to a lack of well-defined clinical characteristics, it is easy to misdiagnose the condition and delay treatment, resulting in a higher disability and death rate, which can cause a serious burden to patients, families, and society (Dong et al., 2019). Currently, up to 85% of encephalitis is unexplained. Although the International Encephalitis Consortium (IEC) defined the diagnostic criteria of encephalitis (Venkatesan et al., 2013), some challenging problems still remain in their clinical application (Granerod et al., 2011; Regner et al., 2018; Bewersdorf et al., 2019), as follows: (i) biopsy represents the “gold standard” for encephalitis; owing to its invasive nature and high false positive rate, it is rarely performed in the clinic; (ii) symptoms are atypical and disease progression is rapid, it is easily confused with other brain disorders, and sometimes a definitive treatment is lacking; and (iii) significant cerebrospinal fluid (CSF) pleocytosis or demonstrable neuroimaging abnormalities are often regarded as surrogate markers of brain inflammation in the absence of pathologic evidence; however, there is a high false-negative rate. In addition to the consensus reached by the IEC, other consensus statements or guidelines have also proposed that imaging technology will play an important role in the early diagnosis of the disease.

Currently, multiple imaging methods are used to improve the diagnosis of encephalitis. Conventional magnetic resonance image (MRI), with the advantages of high soft tissue resolution and the non-invasive nature of the procedure, has been widely used to diagnose encephalitis in the clinic; however, it can only provide anatomical information and the physiological or biochemical information that can be acquired is limited (Zoccarato et al., 2019). Moreover, the perfusion information of lesions can be obtained by injecting gadolinium (Gd)-based contrast agents and, in turn, can increase the risk of Gd deposition and potential side effects (Rogosnitzky and Branch, 2016). Magnetic resonance spectroscopy (MRS) measurement is time-consuming and has poor spatial specificity *in vivo*, which has also limited its applicability despite the fact that it can provide unparalleled opportunities for understanding diseases in terms of metabolism information (Burger et al., 2019). In addition, positron emission tomography (PET) or single-photon emission computed tomography (SPECT) can provide energy metabolism information of diseases, but it has the disadvantages of low specificity, high cost, and the requirement for an intravenous injection of radioactive or ionizing agents (Nishiguchi et al., 2017; Muller Herde et al., 2019). Therefore, there is a need for a non-invasive molecular imaging technology with high spatial resolution that does not require the administration of contrast agents.

Chemical exchange saturation transfer (CEST) is a relatively novel MR molecular imaging approach that utilizes a frequency selective radiofrequency (RF) irradiation pulse on particular exchangeable protons (e.g., hydroxyls, amides, and amines), thus resulting in attenuated water signals that can be measured via the loss of water signal intensity to indirectly characterize the microenvironment of the solution (Ward et al., 2000; Li et al., 2017). CEST MRI has several advantages (Wu et al., 2015; Jia et al., 2019; Wang et al., 2019), as follows: (1) it allows amplified detection of low concentration agents; (2) it can be switched “on” and “off” at will by adjusting the RF irradiation pulse parameters; (3) it has the potential to provide metabolite information from biological tissues as well as anatomical features; (4) it has high spatial resolution, is non-invasive, and does not require the injection of contrast agents; and (5) it can be specifically tailored to respond to a given stimulus (e.g., pH, enzyme, temperature, metabolite levels, etc.). Given these advantages and good performance, CEST MRI has received much attention and is now widely used in preclinical and clinical research. Glutamate (Glu) is the most abundant excitatory neurotransmitter in the brain and is involved in learning, memory, emotion, and cognitive function. The fact that amine protons on Glu that show a chemical shift of 3.0 ppm away from bulk water (0 ppm) can be measured indicates that GluCEST MRI represents a feasible approach for the diagnosis of disease (Cai et al., 2012). Currently, GluCEST imaging has been widely used in psychiatric disorders. For example, Mao et al. (2019) demonstrated that GluCEST imaging could be used for the diagnosis of acute traumatic brain injury and prediction of its prognosis. Bagga et al. (2016, 2018) successfully used GluCEST MRI in a mouse model of dopamine deficiency and Parkinson’s disease to measure spatial changes in Glu. Davis et al. (2015) showed that GluCEST MRI allows the visualization of cerebral Glu changes in rat models of stress-induced sleep disturbance and status epilepticus (Lee et al., 2019). Crescenzi et al. (2014) identified Glu deficits in mouse models of dementia using GluCEST imaging. All of these studies show that GluCEST MRI may represent a valuable approach for interpreting alterations in cerebral biochemical information. Recently, an abundance of evidence has indicated that Glu is implicated in microglial activation and that activated microglia play a key role in encephalitis (Takaki et al., 2012; Zhang et al., 2016). However, the mechanism linking Glu and encephalitis is still unclear.

In this study, we hypothesized that Glu is involved in the occurrence of encephalitis and may serve as a potential biomarker for the diagnosis of incipient encephalitis. Moreover, we hypothesized that GluCEST imaging could be used to predict encephalitis progression or prognosis. Our study may provide new insight into encephalitis and help improve the differential diagnosis of brain disorders.

## Materials and Methods

### Pre-clinical Research

#### Phantom Preparation

Glu (Sigma Aldrich, St Louis, MO, United States) phantoms were first prepared for the optimization of GluCEST MRI parameters. To evaluate whether the CEST effect of Glu was concentration-dependent, different concentrations of Glu (0, 5, 10, 20, 40, and 50 mM) were prepared and their pH was titrated to 7.0. To simulate the effect of other metabolites on GluCEST under physiological conditions, different concentrations of metabolites [*N*-acetylaspartate (NAA; 10 mM), myo-inositol (MI; 10 mM), creatine (Cr; 6 mM), glutamine (Gln; 2 mM), Glu (10 mM), and γ-aminobutyric acid (GABA; 2 mM)] were also used in our study (pH 7.0, room temperature). Before the CEST imaging scan, the nuclear magnetic resonance tubes were inserted into a phantom holder filled with 3% agarose gel to minimize susceptibility inhomogeneity.

#### Animal Model Preparation

All animal care and experimental procedures were approved by the Animal Care and Use Committee of Shantou University Medical College and were in accordance with guidelines from the Chinese Animal Welfare Agency. Twenty-seven adult SD rats, weighing 250–300 g, were used in the experiment and contralateral tissue was collected as a control. All rats were kept in a specific pathogen-free animal room with a temperature-controlled system and a 12-h dark–light cycle. Animals were allowed free access to water and food. *Staphylococcus aureus* (Biotechnology Institute of Beina Chuanglian, Beijing, No. GIM1.160) was cultured overnight on blood agar, with the concentration determined to be 10^7^/μm. Rats were anesthetized with 3∼4.0% isoflurane vaporized with 5% O_2_. The animals were mounted in a stereotactic frame, the rats’ skulls were exposed through a skin incision, and a hole was drilled 4 mm away from bregma in the right side. This hole was situated in the right frontal lobe. Then, 2 μm suspension containing 10^7^/μm *S. aureus* was injected 2.5 mm deep into the hole, with an injection time of 3 min. At pre-injection (0 days) and 3- and 7-day post-injection of *S. aureus*, MR scans were used to observe the dynamic changes of disease in rats.

#### MRI Acquisitions at 7.0 T

All imaging procedures were performed on an Agilent 7.0 Tesla (7.0 T) MR scanner (Agilent Technologies, Inc., Santa Clara, CA, United States) with a standard 9563 body coil for signal transmission and reception. To eliminate signal interference of B_0_ field inhomogeneity, the B_0_ map was corrected prior to the experiments with the following parameters: repetition time (TR) = 40 ms, echo time (TE) = 3, 3.5, and 4 ms, slice thickness = 2 mm, field of view (FOV) = 35 mm × 35 mm, matrix size = 64 × 64, and average = 12. High-resolution T2-weighted axial slices were acquired with TR = 4000 ms, TE = 10 ms, slice thickness = 2 mm, and FOV = 35 × 35 mm. For *in vitro* and *in vivo* experiments, an improved version of continuous wave echo planar imaging sequence was used with the following parameters: TR/TE = 1500/14 ms, saturation power (*B*_1_) = 3.6 μT (*in vitro* phantoms) and 5.9 μT (*in vivo*), saturation time = 2 s, FOV = 35 mm × 35 mm, slice thickness = 2 mm, matrix size = 64 × 64, average = 1. Z-spectra with 52 frequency offsets from -5 to +5 ppm with intervals of 0.2 ppm, and the reference image (S_0_ image) were obtained.

### Clinical Research

#### Human Subjects

This study was approved by the local institutional review board. According to procedures approved by our hospital ethics committee, informed consent was provided before MR examination. Six patients with encephalitis (4 women, 2 men, age 48.17 ± 5.53 years), six patients with lacunar infarction (LI; 3 women, 3 men, age 52.5 ± 4.6 years), and six healthy volunteers (3 women, 3 men, age 46.33 ± 7.31 years) were recruited into this study. According to the major criterion of encephalitis established by the IEC, participants with altered mental status (including decreased level of consciousness, lethargy, or personality change) lasting ≥24 h and/or laboratory examination (lumbar puncture, complete blood count) or electroencephalography consistent with inflammatory changes, were diagnosed with encephalitis. Participants were excluded from the study if any of the following conditions were met: (i) MRI revealed brain injury, hematoma, or tumor; (ii) any contraindication for MRI (e.g., claustrophobia, cardiac pacemaker, or metal in the body); and (iii) any neurological disorder (e.g., schizophrenia, depression) diagnosed by two experienced neurologists and a radiologist, according to clinical symptoms and the MRI scan.

#### MRI Acquisitions at 3.0 T

All MR data acquisitions were performed on a 3.0 T MR system (Sigma; GE Healthcare, Milwaukee, WI, United States) equipped with an eight-channel phased-array head coil. Anatomy images were acquired by a fast spin echo sequence with the following scanning parameters: (1) T2-weighted imaging (T2_W_ imaging): TR = 4480 ms, TE = 120 ms, FOV = 240 mm^2^ × 240 mm^2^, resolution = 256 × 384, and slice thickness = 5 mm; (2) T2_W_ imaging-fluid attenuated inversion recovery (T2Flair): TR = 8600 ms, TE = 155 ms, TI = 2100 ms, FOV = 240 mm^2^ × 240 mm^2^, resolution = 256 × 384, and slice thickness = 5 mm; and (3) diffusion-weighted images (DWI): TR = 6000 ms, TE = min, *b* values = 1,000, FOV = 240 mm^2^ × 240 mm^2^, resolution = 256 × 384, and slice thickness = 5 mm. In addition, a magnetization transfer (MT)-prepared gradient echo MRI sequence was used for CEST imaging with the following parameters: TR = 50 ms, TE = 3.1 ms, FOV = 240 mm^2^ × 240 mm^2^, matrix = 128 × 128, slice thickness = 5 mm, bandwidth = 15.63 kHz. The MT saturation pulse was set to 4 ms width Fermi pulse with flip = 600° (*B*_1_ = 1.95 μT). Forty-one equidistant frequency offsets between 5 and -5 ppm and an additional S_0_ image were acquired. Z-spectra were corrected for B_0_ inhomogeneity using a water saturation shift referencing map (WASSR) with the saturation power and time are 0.1 μT and 20 ms, respectively.

### Image Processing and Data Analysis

All of the CEST image processing and data analyses were performed using custom-written scripts in MatLab (Math works, Natick, MA, United States, R2011b). Z-spectra were calculated from the normalized images for the region of interest (ROI) outlined in each phantom compartment. The GluCEST contrast map, also called the magnetization transfer ratio (MTR_asym_), was defined by the following equation (Cai et al., 2012):

$${\text{MTR}}_{\text{asym}}=\frac{\text{S}\,\left(-3\,\text{ppm}\right)-\text{S}\,\left(+3\,\text{ppm}\right)}{{\text{S}}_{0}}$$

where S (-3 ppm) and S (+ 3 ppm) are the water signal with a saturation pulse at offsets ± 3 ppm from the water resonance, respectively. S_0_ is the water signal without the saturation pulse. The sex and electroencephalogram (EEG) proportions were tested using a chi-squared (χ^2^) test. Age and education were assessed in the three groups using one-way analysis of variance (ANOVA). The MTR_asym_ data, cerebrospinal fluid white blood cell (CSF WBC) count, and disease duration were analyzed using an unpaired *t* test. Statistical evaluations were performed using GraphPad Prism software and differences with *p* < 0.05 were deemed statistically significant.

## Results

### Phantom Studies

**FIGURE 1:**
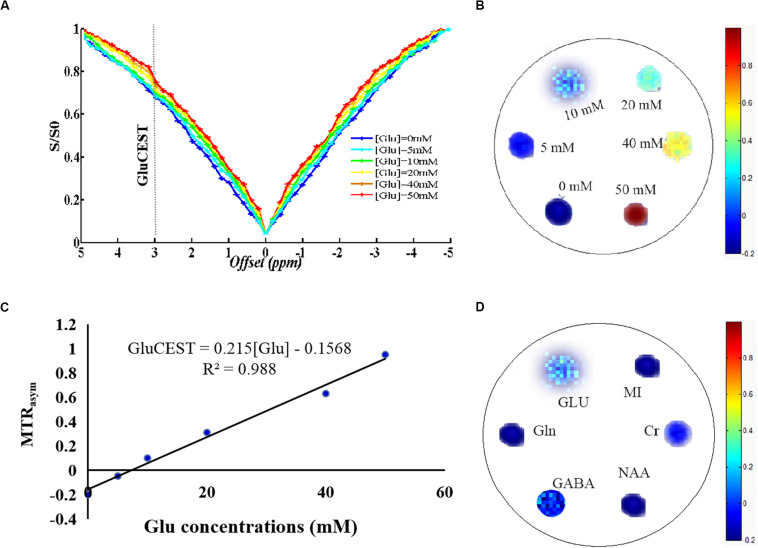
Phantom studies performed on 7.0 T scanner. **(A)** Z-spectra show that the amine protons on glutamate (Glu) generated a chemical exchange saturation transfer (CEST) effect with a chemical shift of 3.0 ppm away from bulk water. **(B)** GluCEST map showing that the GluCEST signal was concentration-dependent. **(C)** GluCEST signal increased as the Glu concentrations increase, which appears to have a good linear relationship, indicating that the GluCEST effect increases by nearly 0.215% with every 1 mM Glu added. **(D)** Under physiological conditions, Glu is the main contributor to the GluCEST signal and γ-aminobutyric acid (GABA) and creatine (Cr) have a small contribution to the GluCEST effect, while the contribution of other metabolites [*N*-acetylaspartate (NAA), myo-inositol (MI), glutamine (Gln)] to the GluCEST effect was negligible.

The Z-spectra show that the amine protons on Glu generated a CEST effect with a chemical shift of 3.0 ppm away from bulk water, and the CEST signal of Glu was increased as the concentrations increased, indicating that the GluCEST signal was concentration-dependent (Figures 1A,B). Figure 1C shows a good linear relationship with the regression equation MTR_asym_(%) =  0.215×(*Gluconcentration*)−0.1568(R^2^ =  0. 988), indicating that the GluCEST effect increases by nearly 0.215% for every 1 mM Glu added. In addition, to simulate the effect of other metabolites on the GluCEST signal under physiological conditions, different concentrations of metabolites [Glu (10 mM), GABA (2 mM), Gln (2 mM), NAA (10 mM), Cr (6 mM), and MI (10 mM)] were used in our study. The results show that Glu is the main contributor to the GluCEST signal, GABA and Cr provide a small contribution to the GluCEST effect, while the contribution of other metabolites (NAA, MI, and Gln) to the GluCEST effect was negligible (Figure 1D). We also evaluated the selection of parameters from the phantoms test and applied the selected optimal parameters to the subsequent *in vivo* application.

### GluCEST Imaging *in vivo* in Encephalitis Model Rats

**FIGURE 2:**
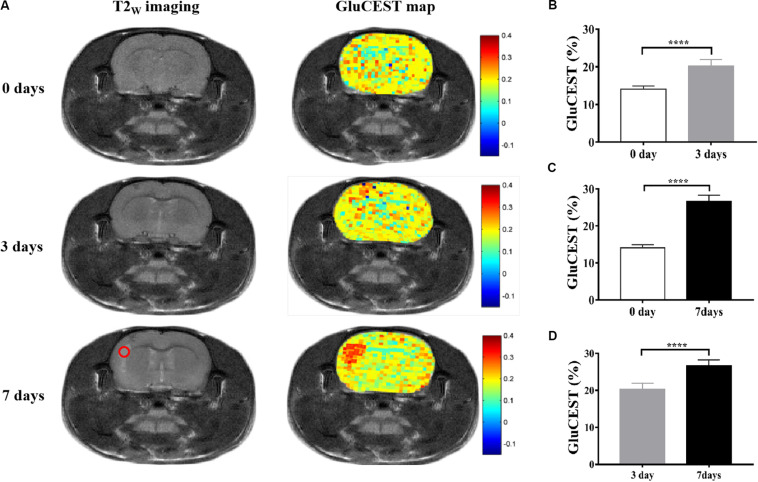
Glutamate chemical exchange saturation transfer imaging *in vivo* in encephalitis rats. **(A)** T2_W_ imaging and GluCEST map of encephalitis rats with *Staphylococcus aureus* infections at different time points (0, 3, and 7 days), suggesting that the GluCEST signal was increased with increasing time; statistically significant differences were found among the different time points [0 days vs 3 days **(B)**, 0 days vs 7 days **(C)**, and 3 days vs 7 days **(D)**, *n* = 27, Student’s *t* test, two-tailed, unpaired, ****p < 0.0001].

All anatomic images clearly showed the presence of encephalitis lesions, suggesting that we successfully established the encephalitis rat model. The GluCEST imaging had high spatial resolution, which could distinguish the encephalitis lesions and separate the white matter and gray matter tissue of rats (Figure 2A). To evaluate whether Glu was involved in the development of encephalitis, GluCEST imaging was used to directly observe Glu *in vivo*. The results show that the mean MTR_asym_ (3.0 ppm) in the encephalitis areas was elevated 3 days after infection with *S. aureus* compared with the healthy control (HC) group (20.24 ± 1.71% vs 14.09 ± 0.79%, *t* = -16.985, *p* = 0.000), indicating that Glu was involved in the occurrence of encephalitis and that GluCEST imaging could be used for the diagnosis of encephalitis (Figure 2B). In addition, to acquire new insight into viable inflammation microenvironments, dynamic changes in Glu concentrations were also observed at different time points (3 and 7 days after infection) in encephalitis rats. The results show that the GluCEST signal was increased with time, and statistically significant differences were found between the various time points [26.66 ± 1.63 vs 14.09 ± 0.79%, *t* = -36.105, *p* = 0.000 (7 days vs 0 days) (Figure 2C); 26.66 ± 1.63 vs 20.24 ± 1.71%, *t* = -14.102, *p* = 0.000 (7 days vs 3 days) (Figure 2D)].

### Demographics and Clinical Characteristics

All subjects successfully completed the experiment. No significant differences were noted among the three groups (encephalitis, LI, and HC) in terms of age (*F* = 1.737, *p* = 0.2097), sex (χ^2^ = 0.45, *p* = 0.799), or education (*F* = 2.807, *p* = 0.0921). Similarly, no statistically significant difference was found in EEG (χ^2^ = 3.086, *p* = 0.079) or disease duration (19.17 ± 2.798 vs 15.0 ± 1.461, *p* = 0.261) between patients with encephalitis and LI. However, the CSF WBC count in the encephalitis group was higher than that in the LI group (9.50 ± 2.432 vs 2.50 ± 0.6708, *p* = 0.0196), indicating that suspected encephalitis patients could be diagnosed with encephalitis without requiring biopsy. The detailed demographic characteristics of subjects are presented in Table 1.

**TABLE 1:** Demographic characteristics of the subjects.

Characteristics	HC group (*n* = 6)	LI group (*n* = 6)	Encephalitis (*n* = 6)	χ^2^/*F*/*t*	*p* value
Sex (M/F)^a^	3/3	3/3	2/4	0.45	0.799
Electroencephalogram (+/-)^a^	–	2/4	5/1	3.086	0.079
Age (years)^b^	46.33 ± 7.312	52.5 ± 4.461	48.17 ± 5.529	1.737	0.2097
Education (years)^b^	9 ± 1.789	7.667 ± 1.211	10.17 ± 2.317	2.807	0.0921
CSF WBC (count/mm^3^)^c^	–	2.50 ± 0.6708	9.50 ± 2.432	2.774	0.0196*
Disease duration (days)^c^	–	15.0 ± 1.461	19.17 ± 2.798	1.32	0.261

*^a^The sex and electroencephalogram (EEG) were tested using a chi-squared test. ^b^Age and education were analyzed using one-way analysis of variance (ANOVA). ^c^ The cerebrospinal fluid white blood cell (CSF WBC) count, and disease duration were analyzed with unpaired *t* test. *p* < 0.05 (^∗^) was considered statistically significant. HC, healthy control; LI, lacunar infarction.*

### GluCEST Imaging *in vivo* in Patients With Encephalitis

**FIGURE 3:**
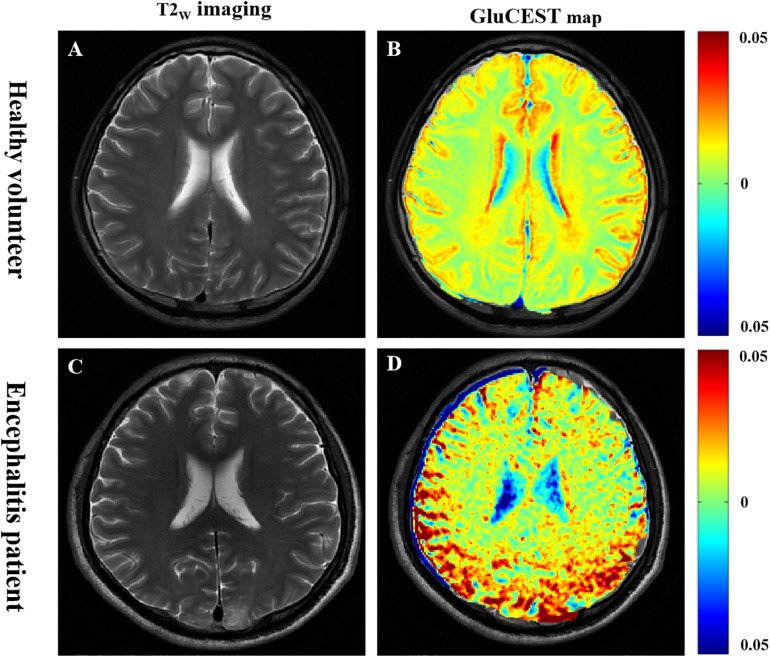
Glutamate chemical exchange saturation transfer imaging was feasible in human brain using the 3.0 T MR system. **(A,B)** In a healthy volunteer, GluCEST magnetic resonance image (MRI) could clearly distinguish white matter and gray matter, similar to T2_W_ imaging, and the gray matter signal intensity of GluCEST was higher than that of white matter. **(C,D)** In a patient with encephalitis, GluCEST imaging also clearly showed the left parietal cortex and subcortex lesion.

**FIGURE 4:**
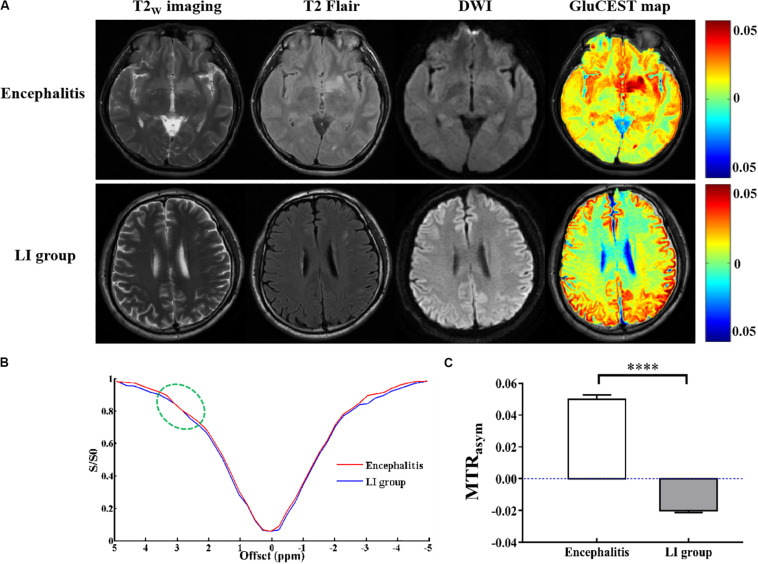
Glutamate chemical exchange saturation transfer imaging *in vivo* of patients with encephalitis and lacunar infarction. **(A)** Conventional T2w imaging, T2Flair, and diffusion-weighted images (DWI) could not discriminate encephalitis and lacunar infarction (LI), but GluCEST MRI could distinguish between them. **(B)** Z-spectrum demonstrated that a small signal dip at 3.0 ppm (green circle) was observed in the inflamed regions but not observed in the LI regions, indicating that inflammation is a cause of the elevated local glutamate concentration. **(C)** GluCEST signal was elevated in patients with encephalitis and decreased in patients with LI (*n* = 6, Student’s *t* test, two-tailed, unpaired, ****p < 0.0001).

As shown in Figure 3, GluCEST MRI could clearly distinguish white matter and gray matter in a similar manner to T2_W_ imaging, and the gray matter signal intensity of GluCEST was higher than that of white matter. In addition, it could clearly detect the lesion area. Hence, using GluCEST MRI to map the distribution of Glu in human brain structures is feasible using a 3.0 T MR system. Compared with the HC group, the signal intensity of GluCEST was elevated in patients with encephalitis, while patients with LI showed a decreased GluCEST signal intensity (Figure 4A). Moreover, a significant difference was found between patients with encephalitis and LI in terms of the GluCEST signal (5.0 ± 0.27 vs -2.0 ± 0.11%, *t* = 59.745, *p* = 0.000) (Figure 4C). Z-spectrums are often used to provide qualitative insights into the CEST imaging exchange mechanism and complete understanding of the physics of the phenomenon. In our study, the average z-spectrum was also drawn based on the ROIs in the lesions. The results demonstrate that a small signal dip at 3.0 ppm was observed in the inflamed regions, but this was not observed in the LI regions, indicating that inflammation may be a cause of elevated local Glu concentrations (Figure 4B).

**FIGURE 5:**
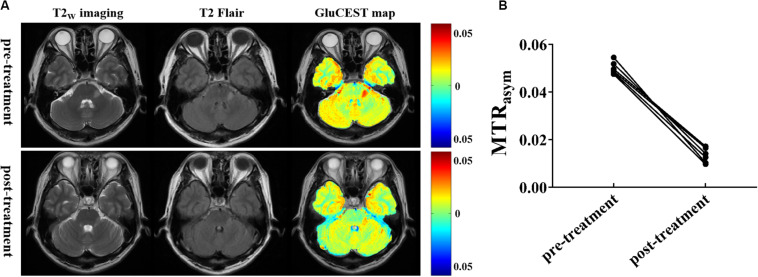
Glutamate chemical exchange saturation transfer imaging was used for efficacy evaluation. **(A)** T2_W_ imaging, T2Flair, and GluCEST maps of a patient with encephalitis before and after intravenous immunoglobulin therapy. **(B)** Compared with pre-treatment, the magnetization transfer ratio (MTR_asym_) value (GluCEST signal) at post-treatment was significantly decreased (*n* = 6, Student’s *t* test, two-tailed, paired).

To explore the relationship between Glu concentration and the severity of lesions, we compared the changes in Glu before and after intravenous immunoglobulin treatment in both patients with encephalitis and LI. In patients with encephalitis, the results show that the signal intensity of GluCEST in encephalitis regions was obviously decreased post-treatment compared with pre-treatment (1.34 ± 0.31 vs 5.0 ± 0.27%, *t* = 20.205, *p* = 0.000), and the range of the signal intensity of the lesions was also reduced in the anatomical images (T2_W_ imaging and T2 Flair) (Figure 5). However, no significant changes were found in either the GluCEST signal or morphology in patients with LI before or after treatment. This suggests that GluCEST imaging may provide new insight into encephalitis and help improve the differential diagnosis of brain disorders and the monitoring of treatment responses.

## Discussion

In this study, we evaluated the *in vivo* application of GluCEST imaging in both a rat model of *S. aureus*-induced encephalitis and in patients with encephalitis, as well as quantified GluCEST signals and evaluated treatment responses. GluCEST maps indicated higher glutamate concentrations in the encephalitis lesions compared with the control group. To date, several studies have demonstrated that Glu serves as the predominant excitatory neurotransmitter in the brain and is involved in learning, memory, emotion, cognition, and energy metabolism (Lancaster, 2016; Nasrallah, 2017). Excessive accumulation of extracellular Glu induces excitotoxicity in CNS neurons, which may lead to various brain disorders, such as epilepsy, schizophrenia, depression, tumors, etc. (Davis et al., 2015; Roalf et al., 2017; Neal et al., 2019; Kaminski et al., 2020). It was also recently reported that glutamate is closely implicated in microglial activation and that activated microglia play a key role in encephalitis. Hence, there is significant interest in developing Glu as validated biomarker to diagnose encephalitis incipiently and precisely.

In the present study, we first explored the factors affecting the GluCEST signal and whether the GluCEST imaging signal was concentration-dependent using phantoms. We determined that glutamate is the main contributing factor to the GluCEST signal under physiological conditions, which is consistent with results from similar previous studies (Cai et al., 2012; Kogan et al., 2013). Subsequently a rat model of *S. aureus*-induced encephalitis was used to simulate the microenvironment of encephalitis, which aids in further understanding the mechanism of encephalitis. Our research team has previously shown that the GluCEST signal (at 3.0 ppm) in *S. aureus*-induced brain lesions was significantly higher than that in healthy controls (Chen et al., 2018); this result was consistent with a previous similar study showing that the extracellular level of Glu increased 12-fold and 30-fold 8 and 20 h, respectively, after staphylococcal infection, as detected by microdialysis (Hassel et al., 2014). This may be due to the activation of microglia, astrogliosis, inflammatory cells, and pro-inflammatory factors, which can lead to the massive release of Glu into the extracellular space, as well as hindered transamination from Glu (Bagga et al., 2016; Igarashi et al., 2017; Thomas et al., 2020). However, an early MRS study showed that the Glu level is decreased in encephalitis (Lentz et al., 2008). This discrepancy might be attributed to the lower resolution of MRS, and the heterogeneous distribution of Glu may lead to its inaccurate detection. In addition, the result was not completely consistent with that of the study of Liu et al., who showed that a broad CEST contrast over a frequency range from 0.5 to 4 ppm, peaking at 2.6 ppm after injection of *S. aureus* and *Clostridium novyi*-NT (*C. novyi*-NT), respectively (Liu et al., 2013, 2018). The reason for the difference in the maximum MTR_asym_ value may be due to the fact that the MT or relaxation properties are significantly different in tissues *in vivo*, which may affect the shape of the Z-spectra (Stanisz et al., 2005). Although other neurotransmitters such as GABA or Cr also increased, their contribution to the GluCEST signals are quite limited based on the phantom results. In this study, we mainly used GluCEST imaging to directly observe the dynamic changes in Glu concentrations in encephalitis lesions, which is of vital importance for understanding the pathogenesis of encephalitis.

Recently, GluCEST imaging has attracted a lot of attention since it is a novel non-invasive MRI technique that has been widely used in the diagnosis of many diseases. For example, Zhuang et al. (2019) demonstrated that GluCEST imaging could be used to diagnose traumatic brain injury using a 7.0 T MR system. Igarashi et al. (2017) successfully used GluCEST MRI to measure spatial changes in Glu in a rat model of ischemia. However, most reports have focused on pre-clinical animal studies with high field strength. Considering the sensitivity of GluCEST and the limitation of the SAR value, few studies can be readily translated to the clinical setting using the GluCEST imaging technique, even though several researchers have proven that GluCEST MRI can be successfully used to map the Glu distribution in human brain or spinal cord structures and it has high reproducibility (Kogan et al., 2013; Nanga et al., 2018). Fortunately, a preliminary study of our research team successfully used 3.0 T MRI scanner to GluCEST imaging for brain injury, compared it with MRS results, and suggested that the observed signals are highly contributed to glutamate; thus, it is feasible to detect the CEST effect from glutamate at 3.0 T (Mao et al., 2019). In our studies, we demonstrated that GluCEST imaging could be translated to the clinic without the need for contrast agents. Compared with the HC group, the signal intensity of GluCEST in patients with encephalitis was increased, while the signal was decreased in patients with LI. The result was inconsistent with that of Igarashi et al. (2017) who indicated that the Glu concentration was increased in the acute focal ischemic lesion, particularly at the border zone. This may be due to the necrosis or loss of neurons caused by occlusion of small blood vessels in LI and there was little or no microglial activation around the lesion since the amount of Glu released was too little to cause changes in the GluCEST signal. However, in acute focal ischemia, a large amount of Glu is released outside of the cells, and there is more microglial activation around the border zone, leading to an increase in the Glu concentration. Notably, the choice of ROI may also affect the quantitative analysis of the GluCEST signal. In addition, the Z-spectrum allows intuitive observation of a small peak at a specific frequency of Glu (3.0 ppm) in encephalitis and was not observed in patients with LI, indicating that encephalitis causes a mass increase in Glu and that the Glu concentration caused by LI was too low to be observed. In short, GluCEST imaging can be used to distinguish encephalitis and LI, in a similar manner to the way in which Liu et al. (2018) showed that bacCEST MRI permits the differentiation of brain abscesses from glioma tumors. Early detection of the response of a brain abscess to an antibiotic treatment is crucial for treatment planning and adjustment. Conventional MRI is unable to directly detect the treatment response, and abscesses may become initially enlarged when the antibiotic treatment is effective. GluCEST imaging has potential clinical utility for monitoring the treatment response before any morphological changes. Our results show that the GluCEST signal in patients with encephalitis was dramatically decreased after intravenous injection of immunoglobulin therapy compared with before therapy. This study demonstrates that GluCEST imaging has the potential as an imaging biomarker for predicting therapeutic outcomes. In patients with LI, however, no obvious changes were found in the GluCEST signal. The reduction in the GluCEST signal in encephalitis lesions after treatment may be due to the fact that the activation of microglia or pro-inflammatory factors was inhibited by immunoglobulin. This result is consistent with a similar previous study that reported a reduced GluCEST signal following ciliary neurotrophic factor (CNTF) injection in the brain, which was attributed to reduced astroglial cellular activity as well as a reduction in the level of glutamine, a precursor of Glu (Carrillo-De Sauvage et al., 2015). Of course, the detailed mechanism of Glu in encephalitis requires further study.

As a method to indirectly detect the solute molecules, CEST imaging often depends on multiple other tissue parameters, including direct water saturation (DS), semi-solid non-specific MT, nuclear overhauser enhancement (NOE) effect, water longitudinal relaxation time (T1w), and nearby overlapping CEST signal influences. Variation of these parameters may cause misinterpretation of the GluCEST signal in encephalitis. Thus, correction of these non-specific factors is necessary. Since the DS effects are symmetric with respect to the water resonance frequency, they can be removed by asymmetry analysis; thus, we used the GluCEST formula S(−3ppm)−S(3ppm)/S_0_ not the S(−3ppm)−S(3ppm)/S(−3ppm) to calculate the GluCEST signal. MT asymmetry is dependent on the applied saturation pulse parameters, and a strong saturation pulse (saturation power >3 μT) with short duration can largely mitigate the confounding effects of MT asymmetry. Thus, in our study, a strong saturation pulse with short saturation times was applied both in the phantom (3.6 μT, 2 s) and *in vivo* (5.9 μT, 2 s) models. In addition, a strong saturated power can reduce the interference of NOE effect. Although several post-processing techniques have been developed to increase the specificity by removing DS, semi-solid MT, NOE effect, and T1w influences, it is still challenging to remove overlapping CEST signals from other potential contaminations. For instance, proteins also have amines of approximately 3 ppm which could contaminate the GluCEST effect. Recently, Zhang et al. performed both *in vitro* and *in vivo* experiments and demonstrated that exchange-dependent relaxation rate (Rex) technology may be a good choice to address this problem (Zhang et al., 2017, 2018). In short, CEST signals are affected by other contamination factors or non-specific parameters, and this needs further study.

Despite the fact that our study confirmed that Glu was involved in the occurrence and development of encephalitis and can be quantitatively evaluated by GluCEST imaging, there are still some limitations in the present study. For example, (i) biopsy as the “gold standard” for the diagnosis of encephalitis, owing to its invasive nature and deep location, and the small scope of lesions limited its used in this study; (ii) the clinical sample sizes are small, and there was no classification of the encephalitis; and (iii) GluCEST imaging was affected by many factors, and there is a lack of unified clinical scanning and diagnostic standards, etc. In spite of these shortcomings, we are also working hard to solve them. For example, we tried our best to achieve accurate diagnosis through combined clinical symptoms, laboratory tests, and experienced neurologists. In terms of the insufficient sample size, we simulated the change in clinical encephalitis using the encephalitis rat model. Of course, we need to continue recruiting patients with encephalitis, and a grading study is also required. Finally, with the development of the MR system hardware and software as well as the efforts of scientists, a standard imaging scheme for clinical practice research will be developed in the future.

## Conclusion

Our study demonstrates that Glu is associated with the early changes in the occurrence and development of encephalitis both in preclinical and clinical applications, and high-resolution GluCEST imaging could be used for real-time, quantitative, and non-invasive monitoring of its dynamic change without requiring the injection of any exogenous imaging contrast agents. In addition, we could use GluCEST imaging to observe the spatial alteration of Glu before and after treatment and then evaluate the therapeutic effect. Therefore, GluCEST imaging has the potential as an *in vivo* imaging biomarker for metabolic and functional changes in the rat model of encephalitis induced by *S. aureus* and patients with encephalitis and could predict encephalitis progress or prognosis with highly clinically translatable value. Importantly, this may provide us new insight into encephalitis and help improve the differential diagnosis of brain disorders. In the future, it will be promising to develop novel agents based on Glu for targeted diagnosis and treatment of encephalitis.

## Data Availability Statement

All datasets generated for this study are included in the article/supplementary material.

## Ethics Statement

The studies involving human participants were reviewed and approved by the local ethics committee of the Second Affiliated Hospital of Shantou University Medical College. The patients/participants provided their written informed consent to participate in this study. The animal study was reviewed and approved by Animal Care and Use Committee of Shantou University Medical College.

## Author Contrbutions

YChen and RWu designed the study. YChen, KG, YCheng, JQ, and HH performed the experiments and collected clinical data. YL and RWa processed images. YJ carried out statistical analysis and drafted the manuscript. YZ and RWu critically reviewed and revised the manuscript. All authors contributed to the article and approved the submitted version.

## Conflict of Interest

The authors declare that the research was conducted in the absence of any commercial or financial relationships that could be construed as a potential conflict of interest.
